# Changes in Heart Rate Variability Recorded in Natural Situation with T-Shirt Integrated Sensors and Level of Observed Behavioral Excitation: A Pilot Study of Patients with Intellectual Disabilities and Psychiatric Disorders

**DOI:** 10.3389/fpsyt.2017.00004

**Published:** 2017-02-01

**Authors:** Julie Palix, Michel Akselrod, Charly Cungi, Fabienne Giuliani, Jérôme Favrod

**Affiliations:** ^1^School of Nursing Sciences, La Source, University of Applied Sciences and Arts of Western Switzerland, Lausanne, Switzerland; ^2^Department of Psychiatry, Centre Hospitalier Universitaire Vaudois, Lausanne, Switzerland; ^3^Ecole Polytechnique Fédérale de Lausanne, Lausanne, Switzerland; ^4^Clinique Belmont, Geneva, Switzerland

**Keywords:** heart rate variability, parasympathetic nervous system, sympathetic nervous system, intellectual disability, psychological stressors

## Abstract

**Background:**

The present study investigates the possibilities of using heart rate variability (HRV) parameters as physiological markers that precede increase in observed behavioral excitation of intellectually disabled individuals. The ability to recognize or predict such patterns, especially in patients showing unpredictable reactions and language deficiencies, might be a major step forward in clinical research.

**Method:**

Thirteen volunteers with intellectual disabilities, who had suffered of at least one event of overt aggression in the preceding 3 months, participated to the study. The protocol consists in the acquisition of continuous electrocardiogram (ECG) throughout approximately two times of 8 h in natural situation, using a T-shirt integrated with sensors. Simultaneously, an observer evaluates the patient’s level of overt excitation from calm (level 1) to extremely tense (level 5) and send online *via* Bluetooth these triggers into the ECG signals. The HRV indexes were then estimated offline on the basis of the inter-beat intervals recorded by the ECG, independently for the 30 min preceding each behavioral tension marking point, averaged, and compared through non-parametric Wilcoxon matched-pairs test. Of these, the RMSSD and LF/HF calculations were used to observe the fluctuations of inhibitory activity and cardiovagal balance through different tension states.

**Results:**

Seven individuals have sufficient reliable data for analysis. They have reached at least a level 3 of behavioral excitation (moderately tense) or more (very to extremely tense, level 4 and 5) and have been retained for further analysis. In sum, a total of 197 periods of tension were kept, made up of 46 periods of slight excitation (level 2), 18 of moderate excitation (level 3), 10 of high excitation (level 4), and 5 of extreme agitation (level 5). Variations in the HRV as a function of degree of excitation are observed for RMSSD index only (inhibitory parasympathetic activity). The changes from calm to increasing levels of excitation are characterized by a significant downfall in RMSSD index when patients were evaluated to be in a very high level of tension (level 4).

**Conclusion:**

The presence of precursors to agitation, reflected in the falling-off of parasympathetic activity, offers potentially interesting prospects for therapeutic development.

## Introduction

The present study is particularly aimed at a population combining mental retardation and psychiatric co-morbidity, with relatively low intelligence quotient (1, 2) and maladaptive behavioral reactions (3). Indeed, such characteristics can lead to inadequate responses to changes in the individual’s environment. The frequency of aggression and abnormal episodes (e.g., self-mutilation, anger, verbal or physical aggression toward others, or property damage) can be a challenge with this population, with a prevalence of 4–10% depending on the country and the duration of observations (4–7). The unpredictability of these behaviors and the deficiencies in individuals’ abilities to communicate their feelings before these episodes take place make them particularly dangerous to themselves and the others.

A person’s ability to deal with situational changes is made possible through the dynamics of his autonomic nervous system (ANS). The ANS either activates or inhibits the body’s system of vigilance through the innervations of the myocardium. This occurs *via* the sympathetic nervous system (SNS) when activating and *via* the parasympathetic nervous system (PNS) when inhibiting. Depending on the psychiatric, psychological, or emotional conditions that an individual is facing, the ANS responses to the demands of their environment could be expressed by in an increase in SNS activity, a decrease of PNS control, or a combination of both (8–11). These autonomic functional differences are described as representative of different emotional state and social position, anxious, and submissive on the one hand with a key behavioral inhibition through the parasympathetic branch (12–16), and hostile and dominant on the other hand, requiring a rapid influx of blood to muscle and the brain through sympathetic-adrenaline activity, which may of may not occur with a lowering of parasympathetic inhibition (17, 18). The ability to recognize or predict of such patterns by examining a patient’s cardiovagal balance, especially in patients showing unpredictable reactions and language deficiencies, might be a major step forward in clinical research to help them. In addition, this type of data might help to clarify the concept of intentionality in the unexpected explosive behavior exhibited by this population.

Educational monitoring such as operant conditioning or applied behavior analysis (19, 20) and breathing control (21, 22) have shown encouraging results in attempts to defuse antisocial behavior and mood fluctuations in long term therapies. A means of recognizing the imminence of aggressive reactions and exploiting that recognition in order to avoid such unpleasant interventions as the seclusion, restraint, or antipsychotic medication of people with an intellectual disability, appears not only essential but also rational and ethical (23–26). Indeed, some heart rate and heart rate variability (HRV) fluctuations have been described to be predictive of reactive aggression (18, 27, 28). This study’s primary goal was to identify markers of increasing physiological agitation—the indicators of imminent aggressive behavior.

## Materials and Methods

### Participants and Methods

Study participants were recruited from patients followed by the mental development psychiatry mobile team of the Community Psychiatry Service at the University Hospital Center of Lausanne, Switzerland, between March and November 2012.

**Figure 1:**
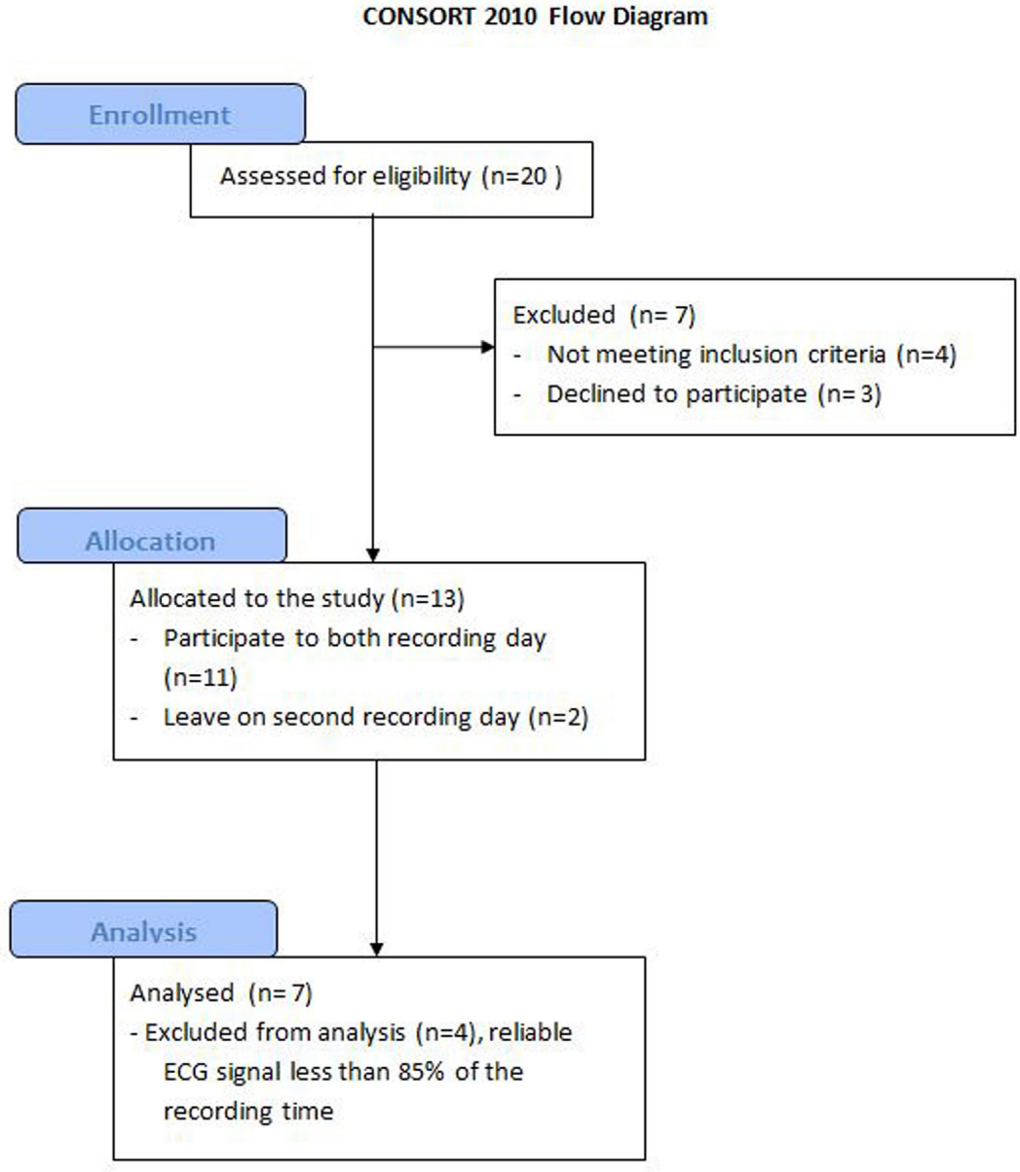
**Consort flowchart**.

The inclusion criteria for participation in this study was at least one recorded episode of aggressive behavior as defined by the Retrospective Modified Overt Aggression Scale (5, 29, 30) in the three months prior to the study (verbal or physical aggression against others, oneself, or property). Each participant wore a T-shirt with integrated sensors developed to record electrocardiograms (Smartex s.r.l., Giuntini 13L Navacchio, Pisa 56023, Italy). This apparatus was designed specifically for the assessment of physiological information in an everyday environment, and it has been successfully tested for the management and study of pathologies such as heart diseases, diabetes, or bipolar disorders (21, 31). A T-shirt was put on after the participant’s morning shower (approximately 9:00 a.m.) and was taken off in the evening (approximately 6:00 p.m.). The T-shirt was worn on two non-consecutive days within 1 week, in order to validate the reliability of the data gathered. Data from the high resolution electrocardiogram (ECG) signal, with sampling every 4 ms (32), were recorded in a device held in the T-shirt’s pocket (CSEM, Neuchâtel, Switzerland). While the ECG signal was recorded continuously, each participant’s “level of excitation” was estimated every half hour by a staff member. Three potential participants refused to be included and two abandoned the study by declining to participate on the second day (see flowchart on Figure 1). Of the remaining 11 subjects, those with a reliable ECG signal for more than 85% of the day were retained, giving a final total of seven participants. The main reason for ECG signal failure was identified as lack of shirt adhesion on the participant’s chest (e.g., because of a BMI below 18, skin diseases, agitation). The final participant group comprised of three women and four men. The average age was 32 years old (SD ± 7.3, min. 28, max. 47). Six participants lived in sheltered accommodation and one lived with parents, three worked in sheltered workshops, six participants took antipsychotic medication, and three took mood stabilizers. As psychoactive substances are recognized as being unrelated to systematic effects on the autonomic system (33, 34), no major recurrence was expected in HRV of our participants as a result of drugs intake. None of the patients were taking a beta-blocker, but one was taking medication for high blood pressure. The seven participants had IQ levels between 35 and 40 to 50 and 55. This study was carried out in accordance with the recommendations of the Human Research Ethics Committee of the Canton de Vaud, Switzerland, and the Declaration of Helsinki. All subjects gave informed written consent to their participation in the study, with the agreement of their legal representative and their family.

### Excitation Levels

During the 8 h of ECG recording, the participant’s level of excitation was estimated by a member of staff every 30 min. A ringtone on a smart phone linked to the recording device *via* Bluetooth reminded the staff member to evaluate the patient. Each participant was evaluated by a single, different evaluator on each day, providing observations of noticeable behaviors representative of different levels of excitation. An observation grid was simplified into five highly distinguishable excitation levels in order to limit inter-evaluator discrepancies: 0, the patient is asleep; 1, the patient is in a calm state; 2, the patient is slightly excited; 3, the patient is moderately excited; 4, the patient is very excited; and 5, the patient is extremely excited. The ringtone acted as a time-based marking point, integrated to the participant’s continuous ECG recording *via* Bluetooth, which could then be matched to each subject’s clinical manifestations of excitation in order to select and analyze of their individual physiological signals corresponding to the clinical manifestations of each subject.

### HRV Measurement

Heart rate variability was estimated independently for the 30 min preceding each behavioral excitation marking point, using the Kubios computing program (Biosignal Analysis and Medical Imaging Group, Department of Physics, University of Kuopio, Finland (35, 36); for an analysis of beat-to-beat variations, and Matlab (MATLAB version 7.10.0, Natick, MA, USA: The MathWorks Inc., 2010) to compute averages for each segment. The variations in beat-to-beat intervals (NN intervals) were extracted and re-sampled using spline interpolation and analyzed in accordance with international guidelines (9, 36–39). Calculations included (1) the square root of the mean of the sum of the squares of the successive differences between adjacent NNs (RMSSD, in milliseconds) and (2) the power of the low frequencies (LF: 0.04–0.15 Hz) and the high frequencies (HF: 0.15–0.4 Hz) after a fast Fourier transform and normalized using logarithmic transformation to calculate the ratio of the low over the high frequencies (LF/HF). The RMSSD index is representative of the inhibitory parasympathetic component of the ANS, whereas the LF/HF ratio is considered to be an indicator of the sympathovagal balance representing an increased in activity in the SNS and/or a reduction in activity in the PNS (9). These two HRV indices were chosen according to previous studies in psychiatric disorders or fragile psychological and emotional states with significant variations (40–43). Furthermore, these indices are frequently used in applied research as shown for mood disorders and emotional-state recognition (21, 22).

### Statistical Analysis

The HRV indices RMSSD and LF/HF have been averaged (mean ± SE) and normalized for each individual (*z*-scores procedure of Statistica, independent across individual data). This procedure is used to shade off individual baseline differences in physiological arousal and thus to improve the examination of fluctuations between tension levels.

## Results

**Figure 2:**
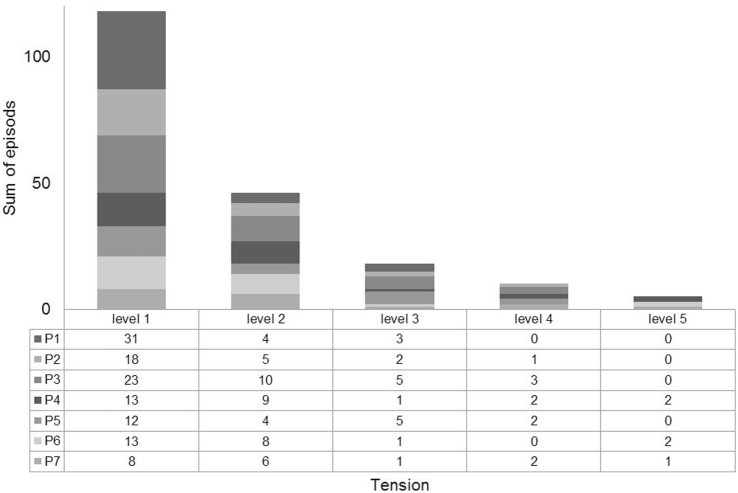
**Detailed contribution of each subject for each tension level (1, calm; 2, slight excitation; 3, moderate excitation; 4, high excitation; and 5, extreme excitation)**.

Seven individuals reached at least a level 3 of overt excitation (moderately tense) or more (very to extremely tense, levels 4 and 5). Among all the marked time periods picked up from the ECG signals, a total of 197 was retained after removing noisy or sleep episodes. In sum, the data of overt tension levels are made up of 46 periods of slight excitation (level 2), 18 of moderate excitation (level 3), 10 of high excitation (level 4), and 5 of extreme agitation (level 5). The detailed contribution of each subject for every tension level is provided in Figure 2.

The physiological values in the calm state (over the seven participants) were measured at 83 b.p.m. (±2 SE) for heart rate frequency, 2.5 (±0.2 SE) for LF/HF ratio, and 68 (±14 SE) for RMSSD index. These estimations are normal to above the expected standard (32, 44).

### HRV Fluctuations and Excitation Levels

**Figure 3:**
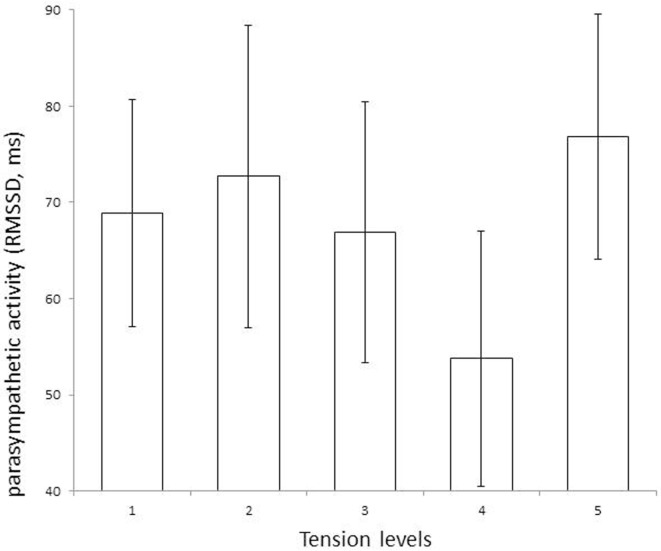
**The parasympathetic inhibitory activity (RMSSD index, Mean, and SE for bars) assessed the change from calm state (tension level 1) to levels of tension 2, 3, 4, and 5**. The RMSSD values decrease from level 2 to 4 (slightly tense to very tense) and enhance at level 5 (extremely tense), suggesting the level 4 to be a key moment of weakness of parasympathetic activity.

Figure 3 shows the variations in the RMSDD variables as a function of degree of excitation. Differences between tension levels are observed for RMSSD index only (inhibitory parasympathetic activity). The change from calm to increasing levels of excitation is characterized by a gradual downfall in RMSSD index with a maximum reduction before the extreme level of tension. Only five occurrences of extreme excitation concerning three participants were observed in this pilot study. The extreme occurrence was measured after outburst. The LF/HF remains unchanged through the different levels of excitation.

## Discussion

This study’s primary goal was to identify markers of increasing physiological agitation that might reveal imminent outburst, particularly in a group of patients with intellectual disabilities and associated behavioral disturbances. By recording continuous ECGs over long periods of time and evaluating the patient’s level of excitation every 30 min, it seems that we identify fluctuations in HRV that might be specific to the moments prior to an increase in the level of excitation. The results of this small pilot study require additional, more in-depth study before they can be confirmed. Nevertheless, we were able to show that the LF/HF ratio did not increase proportionally with excitation. Instead, the index of parasympathetic activity, RMSSD, decreased as the patient’s state changed from slightly tense (level 2) and very tense (level 4), and increased just when reaching extreme excitation (level 5). The increase of RMSSD at level 5 could be explained by the fact that level 5 was utilized when outburst occurred for the three participants concerned. The resolution phase of the outburst might lead to this increase. Thus, this pilot study demonstrated that fluctuations in HRV do not necessarily follow a linear relationship with the observed level of excitation. It also showed that this simple, non-invasive method of collecting ECG data may offer many advantages in efforts to identify changes in physiological arousal prior to potentially aggressive behavior.

Our results show that the RMSSD index measured for the tested population is normal to above the expected standard (32, 44). This normal-to-over-activation of parasympathetic activity discards suggestions that such population exhibit functional parasympathetic failure (12, 13, 45, 46). The deficiencies in behavioral adaptation observed in intellectually impaired subjects could thus be the result of a heightened general state of excitation, with a final breakdown in control when stress reaches an extreme level (30).

Overall, our results seem to suggest that the dominance of the parasympathetic branch of the ANS might demonstrate a position of subordination and a fear of losing control (16, 47, 48). This is similar to recent descriptions related to aggression in fragile X syndrome (49), or brain oscillations of this intellectually disabled population when confronted with emotionally negative images (50). The inhibitory parasympathetic branch appeared late in mammalian evolution in order to suppress the strong fight-or-flight reactions resulting from SNS accelerator inputs (9). Controlling the inhibitory parasympathetic branch is described as promoting interpersonal exchanges and developing social connections (51). According to this pilot study’s results, this nervous inhibitory pathway is mainly activated in individuals with intellectual disabilities and psychiatric conditions, offering new perspectives for therapies that put the emphasis on dealing with anxiety rather than aggressiveness (52). Specific intervention aiming at improving heart rate coherence might be useful. A pilot study using training in heart coherence was tested with mild intellectual disability volunteers working in sheltered workshops. Training in cardiac coherence was implemented during two consecutive weeks. Results indicated that participants could benefit physiologically, psychologically of both (53). However, these results are preliminary and need more controlled validation.

The main limitations of this study are the small sample of participants and the loss of participants due to failure in signal acquisition. The small number of participants prevent from finding a statistically significant result. The ideal design would require a continuous video recording of the behavior of participants to segment signals in realistic fitting periods, but this is difficult to achieve in natural conditions without limiting the freedom of the participants.

In conclusion, the data gathered in this study of a group of patients with intellectual disabilities and psychiatric co-morbidity showing overt aggressive behavior suggests that anxiety-based reactions predominate above hostile aggressive behavior. Furthermore, a decreased in parasympathetic activity during tense condition emerge to be a marker to imminence of agitation.

## Ethics Statement

Commission cantonale d’éthique de la recherche sur l’être humain. Secrétariat administrative, Avenue de Chailly 23, 1012 Lausanne. This study was carried out in accordance with the recommendations of the Human Research Ethics Committee of the Canton de Vaud, Switzerland, and the Declaration of Helsinki. All subjects gave informed written consent to their participation in the study, with the agreement of their legal representative and their family.

## Author Contributions

JF, CC, FG, and JP contributed substantially to the conception and design of the study. JF and FG contributed substantially to data acquisition; JF, MA, and JP contributed substantially to the analysis and interpretation of data. JF and JP drafted the article. MA, CC, and FG revised it critically for important intellectual content. All authors approved the version to be published and agree to be held accountable for all aspects of the work, ensuring that questions related to the accuracy or integrity of any part of it are appropriately investigated and resolved.

## Conflict of Interest Statement

The authors declare that the research was conducted in the absence of any commercial or financial relationships that could be construed as a potential conflict of interest. The reviewer SO and handling Editor declared their shared affiliation, and the handling Editor states that the process nevertheless met the standards of a fair and objective review.
